# Microglia-Derived Extracellular Vesicles Carrying miR-711 Alleviate Neurodegeneration in a Murine Alzheimer’s Disease Model by Binding to Itpkb

**DOI:** 10.3389/fcell.2020.566530

**Published:** 2020-11-06

**Authors:** Yizhi Zhang, Chengbi Xu, Yi Nan, Shanji Nan

**Affiliations:** ^1^Department of Neurology, The Second Hospital of Jilin University, Changchun, China; ^2^Department of Otolaryngology Head and Neck Surgery, The Second Hospital of Jilin University, Changchun, China; ^3^College of Traditional Chinese Medicine, Ningxia Medical University, Ningxia, China

**Keywords:** extracellular vesicles, microRNA-711, Alzheimer’s disease, Itpkb, Tau, microglia

## Abstract

Neurodegeneration in Alzheimer’s disease (AD) results in microglial activation, which may participate in the inflammatory cascade accelerating tissue damage. In this study, we sought to characterize the alleviatory role of microRNA-711 (miR-711) encapsulated in microglia-derived extracellular vesicles (EVs) in a model of AD. Ultracentrifugation was employed to extract EVs from microglia (BV2 cells), which were identified using Western blot analysis of the EVs marker proteins Alix and CD63. A repetitive mild traumatic brain injury (rmTBI) mouse model was induced by controlled cortical impact. After overexpressing miR-711 or 1,4,5-trisphosphate 3-kinase B (Itpkb) in BV2 cells, we evaluated the inflammation in BV2 cells and the ratio of microglia M2/M1. Further, we injected BV2 cell-secreted EVs with overexpressed miR-711 or Itpkb into rmTBI mice through a tail vein to investigate the inflammation markers in mouse serum and, the M2/M1 phenotype ratio of microglia in brain tissues, and to evaluate neurological deficit and cognitive function. The EVs obtained by ultracentrifugation were verified by the presence of Alix and CD63 expression. Mechanistic studies suggested that miR-711 targeted and inhibited Itpkb, thereby repressing Tau phosphorylation and increasing the ratio of M2/M1. Furthermore, miR-711-containing EVs reduced the score of neurological deficits and improved cognitive function in rmTBI mice. The administration of microglia-derived EVs loaded with miR-711, which mediated the hyperphosphorylation of Tau protein in the Itpkb pathway, effectively alleviated neurodegenerative changes and cognitive dysfunction in AD.

## Introduction

Alzheimer’s disease (AD) is a severe neurodegenerative disease, in which neuroinflammation plays a significant role (Gate et al., 2020). As the leading condition contributing to dementia in the old, AD is characterized by intracerebral accumulation of amyloid plaques as well as neurofibrillary tangles (Cortes-Canteli and Iadecola, 2020). The progressive neurodegeneration pathology of AD results in debilitating cognitive impairment (Uddin et al., 2020), which is characterized by dysfunction in learning and memory abilities that are attributed to the neurodegenerative alterations in cerebral cortex and hippocampus (Semba, 2020). Although the molecular pathways underlying the pathogenesis of AD remains uncharacterized, there is considerable evidence that activation of microglia is a factor in the neuroinflammation and degeneration of neurons in the context of AD and other neurological disorders (Rothhammer et al., 2018). Extensive gene- or pathway-based investigations have identified potential biomarkers for improved diagnosis and prognosis in AD (Andrews et al., 2020).

Interestingly, microglia are main endogenous immune cells capable to modulate neurodegenerative reactions in the central nervous system (Rawlinson et al., 2020). Pro-inflammatory microglia (the M1 phenotype) are recognized to augment neuronal damage by producing reactive oxygen species, while the anti-inflammatory microglia (M2 phenotype) can rescue neurons from injury by accelerating wound healing and tissue remodeling (Kobayashi et al., 2013; Plastira et al., 2016). In the central nervous system, extracellular vesicles (EVs) are critically important for the intercellular communication between neurons and microglia (Surgucheva et al., 2012; Paolicelli et al., 2019). EVs secreted by microglia have been unveiled to participate in the functioning of inflammatory pathways (Yang et al., 2018), in their capacity as mediators of cellular communication by shuttling microRNAs (miRNAs), proteins, and messenger RNAs (mRNAs) (Tesovnik et al., 2020).

EV-mediated delivery of miRNAs is reported to attenuate neurodegeneration and restore cognitive function in a repetitive mild traumatic brain injury (rmTBI) model that simulates aspects of AD (Ge et al., 2020). It is noted that miR-711 has been identified to modulate anti-inflammatory pathways and M2-like activation phenotype, as was demonstrated previously in the context of neuroinflammatory reactions revealed by microarray expression profiling (Freilich et al., 2013). In addition, miRNAs and EV-loaded miRNAs, including miR-711, have been suggested to impact the neuro-immunological interactions between macrophages and sensory neurons (Chen et al., 2019). In this study, the binding affinity between miR-711 and 1,4,5-trisphosphate 3-kinase B (Itpkb) was predicted by an initial bioinformatics-based analysis. Prior evidence has identified high expression of Itpkb in AD, and linked this expression to enhanced Tau phosphorylation (Salta et al., 2016). Furthermore, the increasing phosphorylation of Tau is a key factor in the cognitive dysfunction in AD (Zheng et al., 2020). Therefore, we aimed in this study to test a hypothesized mechanism of action whereby microglia-derived EVs loaded with miR-711 alleviate against neurodegeneration and cognitive decline in a controlled cortical impact (CCI)-induced rmTBI mouse model, in which Itpkb-dependent Tau phosphorylation is a likely factor in the neuropathology.

## Materials and Methods

### Ethics Statement

Animal experiments were performed in accordance with *Guide for the Care and Use of Laboratory Animals* published by the National Institutes of Health, and approved by the Animal Ethics Committee of Jilin University. All efforts were made to minimize the discomfort and number of experimental animals.

### Experimental Animals

C57BL/6 mice (male, 8 weeks old) were purchased from the Shanghai Slac Laboratory Animal, Co. Ltd. (Shanghai, China) and were housed in the specific pathogen free animal room for acclimation during at least 1 week. The animal quarters were kept at a temperature of 20–22°C with relative humidity of 40–60%, and a 12-h light/dark cycle. Animals were provided with access to food and water *ad libitum*. For inducing the rmTBI model, we constructed a molded acrylic casting to fix the mice in place, provided with a 3.0 mm space for head acceleration/deceleration below the impact point.

### CCI-Induced rmTBI Mouse Model

This model initiates a process of chronic neurodegeneration leading to cognitive dysfunction within 5–6 weeks after injury. After inducing anesthesia with 4.6% isoflurane, the mice were fixed in the custom-made hold, with a 3 mm space for head motion upon impact. The impact was applied using an Electronic CCI (American Instruments, Richmond, VA, United States) with an impact velocity of 5 m/s and a horizontal transit of 2.0 mm. After receiving an impact, the mice were placed in a well-ventilated cage at 37°C until they regained consciousness. The impact was repeated four times, at intervals of 24 h. Twenty-four hours after the last impact, follow-up experiments were conducted.

### Bioinformatics Analysis

The important miRNAs related to AD are determined by existing literature, and the downstream genes of miRNA are predicted using TargetScan^1^. R language “limma” package^2^, with —logFoldChange— > 1 and *p* < 0.05 as the threshold, was employed to analyze AD-related expression profile GSE5281 in the Gene Expression Omnibus (GEO) database^3^. The expression profile GSE5281 contains 161 samples, including 74 normal samples and 87 AD samples. The intersection of miRNA downstream genes and the top 15 differentially expressed genes was shown using a Venn diagram to identify important downstream genes. String^4^ was used to predict related genes of important downstream genes and construct a protein-protein interaction (PPI) network. Cytoscape^5^ was introduced to select the genes with the highest core degree as the downstream genes for further study. The binding site of miRNA and gene was obtained from TargetScan, and the expression trend of the gene was confirmed by the data of expression profile GSE5281.

### Cell Culture

Mouse glial cells (BV2 cells) and HEK293T cells, purchased from the American Type Culture Collection (Rockville, MD, United States), were cultured in Dulbecco’s Modified Eagle Medium (Gibco, Carlsbad, CA, United States) containing 10% fetal bovine serum (FBS) and 1% penicillin. Cells were passaged three times a week at a ratio of 1:3. The cells were cultured in an incubator at 37°C in 5% CO_2_.

### Separation and Identification of EVs

EVs were separated from the culture supernatant by ultracentrifugation. The collected BV2 cell culture medium was centrifuged at 2000 × *g* for 10 min at 4°C. The collected supernatant was then centrifuged at 10,000 × *g* for 10 min at 4°C. The supernatant was filtered through a 0.2 μm filter (Steradisc, Kurabo, Osaka, Japan) to remove bacteria, and then centrifuged at 100,000 × *g* for 70 min at 4°C (Beckman, Brea, CA, United States). The pellet was washed with phosphate buffer saline (PBS), and then centrifuged at 100,000 × *g* at 4°C for 70 min. The enriched pellet was resuspended in sterile PBS for subsequent studies. EVs were identified using transmission electron microscopy (TEM). EVs (20 μL) were added dropwise on the copper grid net and allowed to stand for 3 min. The liquid was blotted dry with the edge of a filter paper, and 30 μL of phosphotungstic acid solution (pH = 6.8) was added dropwise. After counterstaining at room temperature for 5 min, EVs were dried under an incandescent lamp and then examined by TEM. The particle size was determined using nanoparticle tracking analysis (NPTA). The expression of specific marker proteins CD63 (sc-5275, 1:200, Santa Cruz Biotechnology Inc., Santa Cruz, CA, United States) and Alix (sc-53540, 1:200, Santa Cruz) on the surface of EVs was measured by Western blot assay, with Calnexin (ab92573, 1:2000, Abcam, Cambridge, United Kingdom) as negative control (NC).

### Cell Transfection

miR-711 mimic and mimic NC were purchased from GenePharma, Co. Ltd. (Shanghai, China). The transfection procedure was performed according to the instructions of the Lipofectamine 2000 kit (cat #11668500, Invitrogen, Carlsbad, CA, United States). Both miRNA and Lipo2000 were separately dissolved in opti-Minimal Essential Medium, and the two solutions were gently mixed. After standing at room temperature for 20 min, the mixture was slowly added dropwise into BV2 cells. The final miRNA concentration was 50 nM.

### Lentivirus Infection

The overexpression (OE)-Itpkb lentivirus (GeneChem, Co. Ltd., Shanghai, China) with a virus titer of 10^9^ infectious units was infected into BV2 cells according to the manufacturer’s instructions, and was designated as NC-BV2 cells and OE-Itpkb-BV2 cells. After 24 h of infection, follow-up experiments were conducted.

### Flow Cytometry

The mouse brain tissues were detached with collagenase and passed through a 70 μm nylon mesh to obtain a single cell suspension. BV2 cells were seeded in 10 cm Petri dishes and treated according to their experimental grouping. In brief, a single cell suspension was reacted with allophycocyanin-bound CD206 (141-708, Thermo Fisher Scientific, Waltham, MA, United States) (M2 microglia biomarker), and fluorescein isothiocyanate-bound D86 (105110, Thermo Fisher) (M1 microglia biomarker), followed by incubation at room temperature for 45 min. Cells were detected using an LSRFortessa^TM^ cell analyzer (BD Bioscience, San Jose, CA, United States), and samples were analyzed using Flowjo software (Tree Start, Ashland, OR, United States). All antibodies used for flow cytometry were purchased at Santa Cruz.

### Dual-Luciferase Reporter Gene Assay

miR-711 mimic or mimic-NC was co-transfected into HEK293T cells by Lipo2000 with a luciferase reporter plasmid psiCkeck2-Luciferase Reporter vector (Promega, Corp., Madison, WI, United States) containing wild-type (WT) Itpkb 3′ untranslated region (3′ UTR) or mutant (MUT) Itpkb 3′ UTR. The relative luciferase activity of each group of cells was measured 48 h after transfection with the dual luciferase reporter gene detection kit (E1910, Promega). Cells were transfected with empty luciferase reporter gene plasmid, which served as the NC cells. Luciferase Assay Reagent II was added to the samples to detect the intensity of firefly luminescence, after addition of Stop & Glo^®^ reagent to stop the reaction. At the same time, the Renilla luciferase reaction was conducted, and the ratio of firefly luciferase to Renilla luciferase was calculated as the relative luciferase activity.

### Western Blot Assay

Total protein in cells or brain tissues was extracted using phenylmethanesulfonyl fluoride-containing Radio Immunoprecipitation Assay lysis buffer (R0010, Solarbio Science & Technology, Co., Ltd., Beijing, China) pre-cooled at 4°C. The bicinchoninic acid Protein Assay Kit (23227, Pierce, Thermo Fisher) was employed to determine the protein concentration of each sample. After protein separation by polyacrylamide gel electrophoresis, the protein was transferred to polyvinylidene fluoride membranes (Millipore, Billerica, MA, United States) by the wet transfer method, which was then blocked in 5% bovine serum albumin at room temperature for 1 h. The blots were probed at 4°C overnight with diluted primary antibody (rabbit antibodies) to Tau (ab32057, 1:1000, Abcam), phosphorylated-Tau (p-Tau) (ab109390, 1:1000, Abcam), Itpkb (PA5-101099, 1:1000, Thermo Fisher), and glyceraldehyde-3-phosphate dehydrogenase (GAPDH) (ab8245, 1:5000, Abcam). The membrane was rinsed three times with Tris Buffered saline Tween (each time for 10 min), and incubated with horseradish peroxidase-labeled goat anti-rabbit immunoglobulin G (ab6721, 1:5000, Abcam) at room temperature for 1 h. Immunoreactive bands were visualized and quantified using Quantity One v4.6.2 software. Quantitative protein analysis was performed based on the ratio of the gray value of each protein to the gray value of internal reference GAPDH.

### RNA Extraction and Reverse Transcription Quantitative Polymerase Chain Reaction (RT-qPCR)

Total RNA was extracted using Trizol reagent (15596026, Invitrogen). MiRcute miRNA First-strand cDNA synthesis kit (Tiangen Biotech, Beijing, China) was used for RT of miRNA, and Primer-Script^TM^ one step RT-PCR kit (Takara, Shiga, Japan) for RT of mRNA. The synthesized cDNA was subjected to RT-qPCR detection using Fast SYBR Green PCR kit (Applied Biosystems, Foster City, CA, United States) and 7500 Fast Real-Time PCR System (Applied Biosystems). The relative expression of genes was analyzed using the 2^–Δ^ ^Δ^ ^*Ct*^ method. The primer design is shown in Table 1.

**TABLE 1:** Primer sequences for RT-qPCR.

**Gene (m)**	**Primer sequence (5′–3′)**	
GAPDHGapdh	F: AATGGATTTGGACGCATTGGT	R: TTTGCACTGGTACGTGTTGAT
miR-711	F: GGGCTTACATCTCTAAAG	R: CAGTGCGTGTCGTGGAGT
U6	F: CTCGCTTCGGCAGCACA	R: AACGCTTCACGAATTTGCGT
Itpkb	F: GGCTGAATAGTAGCAGCGGTA	R: CTGGTTCACCTGCACATTTTG

*RT-qPCR, reverse transcription quantitative polymerase chain reaction; F, forward; R, reverse; GAPDH, glyceraldehyde-3-phosphate dehydrogenase; miR, microRNA; Itpkb, 1,4,5-trisphosphate 3-kinase B.*

### Enzyme Linked Immunosorbent Assay (ELISA)

BV2 cell culture supernatant and rmTBI mouse serum were obtained. ELISA detection was performed according to the instructions of tumor necrosis factor-α (TNF-α) (PT512, Beyotime Biotechnology, Co. Ltd., Shanghai, China) and interleukin-10 (IL-10) (PI522, Beyotime) kits. The absorbance value was measured with a microplate reader (BS-1101, DeTie Experimental Equipment, Co. Ltd., Nanjing, China) at 492 nm after zero adjustment with a blank control well. The content of the factor to be tested was determined on the standard curve based on the absorbance value of each sample.

### Experimental Grouping

The mice were designated according to two different groupings of (*n* = 12) mice, as follows.

Grouping method 1: NC mimic (BV2 cells transfected with NC mimic) and miR-711 mimic groups (BV2 cells transfected with miR-711 mimic). Here, the EVs from BV2 cells were designated as NC-EVs and miR-711-EVs.
Grouping method 2: sham (operated rmTBI mice without CCI induction), rmTBI (rmTBI mice injected with PBS of equal volume), NC-EVs (rmTBI mice injected with NC-EVs via a tail vein), and miR-711-EVs (rmTBI mice injected with miR-711-EVs via a tail vein). Starting 24 h after the model establishment, 40 μg portions of EVs were injected into a tail vein every 3 days.
Grouping method 3: NC mimic-EVs + OE-NC (rmTBI mice injected with NC mimic-EVs and lentivirus harboring OE-NC via tail vein), NC mimic-EVs + OE-Itpkb (rmTBI mice injected with NC mimic-EVs and lentivirus harboring OE-Ttpkb via tail vein), and miR-711-EVs + OE-Itpkb (rmTBI mice injected with miR-711 mimic-EVs and lentivirus harboring OE-Ttpkb via tail vein) groups. EVs (40 μg) were injected into a tail vein every 3 days starting at 24 h after model development.
Grouping method 4: dimethylsulfoxide (DMSO)-OE-NC (BV2 cells infected with lentivirus harboring OE-NC, and added with DMSO of equal amount), DMSO-OE-Itpkb (BV2 cells infected with lentivirus harboring OE-Itpkb, and added with DMSO of equal amount), and WZ4003-OE-Itpkb [BV2 cells infected with lentivirus harboring OE-NC, and added with 10 μM Tau phosphorylation inhibitor WZ4003 (S7317, Selleck Chemicals, Huston, TX, United States)] groups.

### Modified Neurological Severity Score (mNSS)

Before treatment, rmTBI mice were evaluated for neurological functions by multiple indicators (Table 2) including tests of sensor function, movement, and reflex/balance tests. Higher scores indicated more severe neurological deficits. The measurements were made on days 1, 3, 7, 14, 21, and 28 respectively.

**TABLE 2:** Neurological deficit scores.

**Function studied**	**Score**
***Movement test***	
**Tail suspension test**	
Forelimb flexion	1
Hindlimb flexion	1
Head deviation from the vertical axis within 30s > 100	1
**Ground walking test**	
Able to walk normally	0
Unable to walk straight	1
Circle to the side of paraplegia	2
Fall to the side of the paralysis	3
**Sense test**	
Placement test (vision and touch test)	1
Proprioception test (deep sensation, pressing the mouse paws against the edge of the table to stimulate limb muscles)	1
**Balance beam test**	
Stable and balanced posture	0
Grasp the edge of the balance beam	1
Grasp the balance beam, with one limb falling from the balance beam	2
Hold the balance beam tightly, with two limbs falling from the balance beam, or rotate on the balance beam (>60 s)	3
Trying to balance on the balance beam but falling (>40 s)	4
Trying to balance on the balance beam but falling (>20 s)	5
No attempt to balance on the balance beam (<20 s)	6
**Loss of reflex and abnormal movement**	
Auricle reflex	1
Corneal reflex	1
Panic reflex	1
Epilepsy, myoclonus, dystonia	1

### Morris Water Maze (MWM)

The mice in each group underwent a spatial navigation test 28–32 days after modeling. A round tank (122 cm in diameter and 55 cm in height) filled with opaque water at 22 ± 2°C to a depth of 42 cm was divided into four quadrants. The target platform with a diameter of 15 cm was submerged 2 cm below the water surface in the middle of the quadrant I. The tracking camera was placed above the MWM and used to record the swimming trajectory of each mouse. One of any four quadrants (I, II, III, and IV) was selected to introduce the mouse into the maze, facing the pool wall. Computer software was employed to record the swimming path of each mouse for 90 s as it attempted to locate the hidden platform. The mice that successfully found the platform were allowed to rest on the platform for 10 s. If a mouse could not locate the platform within 90 s, it would be manually placed on the platform for 10 s to allow spatial memory formation. After swimming, the mice were warmed under a 150 W incandescent lamp for 5 min and returned to their home cages. Each mouse was trained four times a day at intervals of 20 min.

### Statistical Analysis

SPSS 21.0 software (IBM, Corp., Armonk, NY, United States) was used for statistical analysis. The measurement data were summarized as mean ± standard deviation. Unpaired *t*-test was used for two-group comparisons. One-way analysis of variance (ANOVA) was carried out for comparisons among multiple groups followed by Tukey’s *post hoc* test. Repeated measures ANOVA with Bonferroni *post hoc* test was employed to compare data at different time points. A value of *p* < 0.05 indicated significant difference.

## Results

### miR-711 Mediates the Anti-Inflammatory Pathway and M2-Like Activation Phenotype of Microglia

**FIGURE 1:**
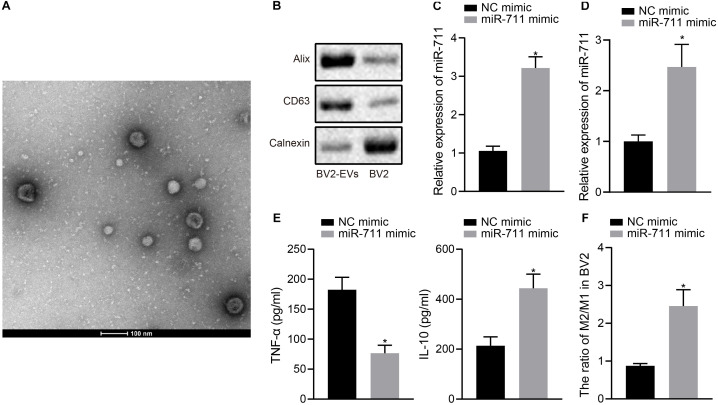
miR-711 mediates the anti-inflammatory pathway and M2-like activation phenotype of microglia. **(A)** Transmission electron microscopy observation of the structure of BV2 cell-secreted EVs and use of the NPTA method to detect the diameter of EVs. **(B)** Western blot analysis to detect the protein expression of Calnexin, Alix and CD63 in EVs derived from BV2 cells and BV2 cells. **(C)** RT-qPCR to detect the expression of miR-711 in BV2 cells. **(D)** RT-qPCR to detect the expression of miR711 in EVs derived from BV2 cells. **(E)** ELISA to detect the expression of inflammatory factors TNF-α and IL-10 in BV2 cells. **(F)** Flow cytometry to detect the content of M2 phenotype and M1 phenotype microglia in BV2 cells, with the ratio calculated. *p < 0.05 versus the NC mimic group. The measurement data were summarized as mean ± standard deviation. Unpaired *t-*test was used for two-group comparisons. Cellular experiment was repeated three times independently.

We validated the function of miR-711 in BV2 cells and performed *in vitro* experiments with BV2 cell-derived EVs. TEM and NPTA detection showed that most of the EVs derived from BV2 cells were round or elliptical in shape, with a mean particle diameter of 98 ± 24.1 nm (Figure 1A). In addition, Western blot assay revealed that, compared to intact cells, the levels of CD63 and Alix protein were higher in EVs, but Calnexin was hardly expressed (Figure 1B). After transfection of miR-711 mimic in BV2 cells, RT-qPCR was performed to measure miR-711 expression in BV2 cells and EVs derived from BV2 cells, showing that miR-711 mimic caused an increase in miR-711 expression compared to the treatment with NC mimic (*p* < 0.01) (Figures 1C,D). ELISA detection of the expression of inflammation factors in the medium showed that miR-711 mimic reduced the expression of TNF-α and increased the expression of IL-10 (*p* < 0.05) (Figure 1E). Forty-eight hours after transfection with miR-711 mimic, BV2 cells were collected and the contents of M1 and M2 microglia was measured by flow cytometry. Results showed that miR-711 mimic treatment increased the M2/M1 ratio of microglia (*p* < 0.01) (Figure 1F). The above results indicate that microglia-derived EVs loaded with miR-711 mediated anti-inflammatory pathway and M2-like activation phenotype.

### Microglia-Derived EVs Relieve Inflammation, Neurodegeneration, and Cognitive Dysfunction in Mice by Delivering miR-711

**FIGURE 2:**
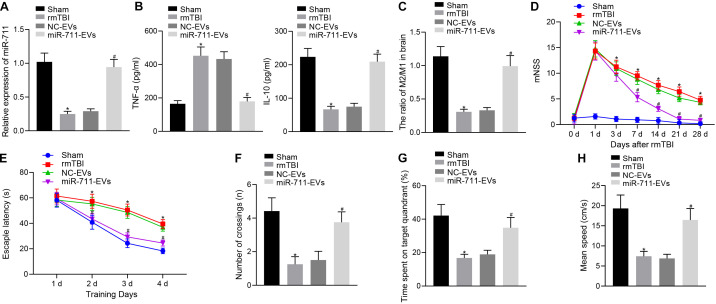
Microglia-derived EVs relieve inflammation, neurodegeneration, and cognitive dysfunction in mice by delivering miR-711. **(A)** RT-qPCR to detect the expression of miR-711 in the brain tissues of mice in each group. **(B)** ELISA to detect the expression of inflammatory factor TNF-α and anti-inflammatory factor IL-10 in mouse serum. **(C**) Flow cytometry to detect the contents and ratio of M1 and M2 microglia phenotypes in mouse brain tissue. **(D)** After the model was successfully constructed, the mice were scored for neurological deficits on days 1, 3, 7, 14, 21, and 28. **(E)** Quantitative analysis of the escape latency of the mice in each group within 4 days of training in the Morris water maze. **(F)** Quantitative analysis of the number of times that the mice of each group crossed the target platform. **(G)** Quantitative analysis of the percentage of time spent by the mice in the target quadrant. **(H)** Quantitative analysis of the average swimming speed of mice in each group. *p < 0.05 versus the Sham group; #*p* < 0.05 versus the NC-EVs group. The measurement data were summarized as mean ± standard deviation. One-way ANOVA was carried out for comparisons among multiple groups followed by Tukey’s *post hoc* test. Repeated measures ANOVA with Bonferroni *post hoc* test was employed to compare data at different time points. *n* = 12.

Following model development, rmTBI mice were injected with 40 μg BV2 cell-secreted EVs via a tail vein. After 14 days, the animals were deeply anesthetized for collection of blood and brain tissues. Analysis of the brain tissues of rmTBI mice showed decreased miR-711 expression, whereas treatment with miR-711-EVs increased the expression of miR-711 (*p* < 0.01) (Figure 2A). ELISA assay of serum samples showed that rmTBI mice had increased TNF-α expression and decreased IL-10 expression, while miR-711-EV treatment decreased TNF-α expression and increased IL-10 expression (*p* < 0.01) (Figure 2B). Mouse brain tissues were prepared into a single cell suspension, and flow cytometric analyses showed that the ratio of microglia M2/M1 in rmTBI mice decreased, while miR-711-EV injection caused an increase in the ratio of microglia M2/M1 (*p* < 0.01) (Figure 2C). In addition, evaluation of neurodegenerative changes in mice revealed that rmTBI mice had increased mNSS scores, while miR-711-EV injection caused a relative decrease in mNSS score (*p* < 0.05) (Figure 2D). From the 28th day after rmTBI development, the spatial navigation test and spatial probe test were employed to evaluate the spatial learning and memory abilities of mice for four consecutive days. During the spatial navigation test, the escape latency of all experimental groups showed a reduction with increasing number of training day, i.e., spatial learning. Relative to sham-operated mice, rmTBI mice presented with prolonged escape latency, while miR-711-EV injection shortened the escape latency (*p* < 0.05) (Figure 2E). During the spatial probe test, the number of times that the rmTBI mice traversed the target platform, the percentage of time spent in the target quadrant and the average swimming speed were reduced relative to sham-operated mice. On the other hand, the miR-711-EV treatment increased the number of times crossing the target platform, the percentage of time spent in the target quadrant, and the average swimming speed (*p* < 0.01) (Figures 2F–H). The above results indicate that microglia-derived EVs can alleviate inflammation, neurodegenerative changes and cognitive dysfunction in mice by delivering miR-711.

### miR-711 Targets and Negatively Regulates Itpkb

**FIGURE 3:**
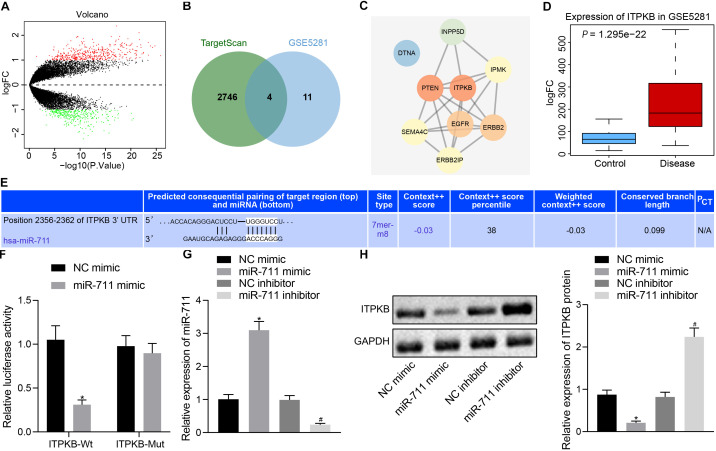
miR-711 targets and negatively regulates Itpkb. **(A)** The volcano expression graph of the expression profile GSE5281. Red dots indicate upregulated genes and green dots indicate downregulated genes. **(B)** TargetScan prediction of downstream genes of miR-711 and Venn diagram of the top 15 genes of expression profile GSE5281. The four genes in the intersection are ERBB2IP, SEMA4C, Itpkb, and DTNA. **(C)** PPI network diagram of the four intersections and their related genes constructed by String. A higher gene core degree corresponds to a darker circle color, whereas a lower core degree corresponds to a bluer color. **(D)** Itpkb expression box plot drawn based on the data of expression profile GSE5281. The blue box on the left indicates the expression in normal samples, and the red box on the right indicates the expression in AD samples. **(E)** The possible binding site of miR-711 to Itpkb predicted by TargetScan. **(F)** Dual-luciferase reporter gene assay to verify whether miR-711 can target Itpkb. **(G)** RT-qPCR to detect the expression of miR-711 in BV2 cells in each group. **(H)** Western blot analysis to detect the expression of Itpkb normalized to GAPDH in BV2 cells in each group. *p < 0.05 versus the NC mimic group; #*p* < 0.05 versus the NC inhibitor group. The measurement data were summarized as mean ± standard deviation. Unpaired *t*-test was used for two-group comparisons. One-way ANOVA was carried out for comparisons among multiple groups followed by Tukey’s *post hoc* test. Cellular experiment was repeated three times independently.

We predicted the target genes of miR-711 through TargetScan, which revealed 2,750 potential target gene genes. We also analyzed the expression profile GSE5281 in the GEO database. In Figure 3A, the red dots indicated upregulated genes and the green dots indicated downregulated genes. Through comparison of the 15 genes with highest significance and the 2750 potential target genes of miR-711, we identified four genes likely to be important (ERBB2IP, SEMA4C, Itpkb, and DTNA) (Figure 3B). The related network genes of these four genes were predicted by String, and the PPI network was constructed. Cytoscape was employed to draw a diagram and calculate the core degree of the gene, which revealed Itpkb to be the gene with the highest core degree (Figure 3C). An expression profile GSE5281 data showed that Itpkb was highly expressed in AD (Figure 3D). TargetScan prediction provided the possible binding sites of miR-711 to Itpkb (Figure 3E). Dual-luciferase reporter gene assay found that, compared with the mimic NC group, Itpkb-WT/miR-711 mimic co-transfection decreased luciferase activity (*p* < 0.01); while Itpkb-MUT/miR-711 mimic co-transfection did not affect luciferase activity, thus suggesting that miR-711 can specifically bind to the Itpkb gene (Figure 3F). In BV2 cells, miR-711 mimic promoted miR-711 expression while miR-711 inhibitor suppressed miR-711 expression (*p* < 0.01) (Figure 3G). Western blot assay revealed that miR-711 mimic decreased Itpkb expression while miR-711 inhibitor increased Itpkb expression (*p* < 0.01) (Figure 3H). The above results indicate that miR-711 may play an important role in AD by targeting Itpkb.

### miR-711 Mediates Anti-Inflammatory Pathway of Microglia and M2-Like Phenotype by Targeting Itpkb

**FIGURE 4:**
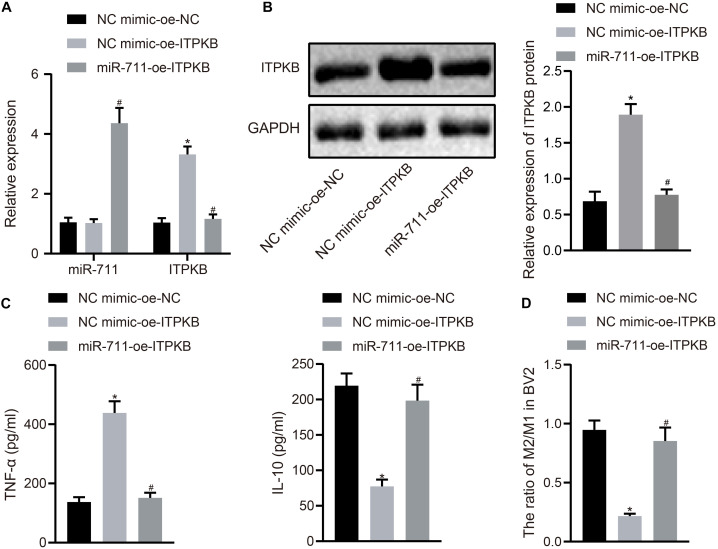
miR-711 mediates anti-inflammatory pathway of microglia and M2-like phenotype by targeting Itpkb. **(A)** RT-qPCR to detect the expression of miR-711 and Itpkb in each group of BV2 cells. **(B)** Western blot assay to detect the expression of Itpkb normalized to GAPDH in each group of BV2 cells. **(C)** ELISA to detect the expression of inflammatory factor TNF-α and anti-inflammatory factor IL-10 in BV2 cell culture medium. **(D)** Flow cytometry to detect the content of M2 phenotype and M1 phenotype microglia in BV2 cells, with the ratio calculated. *p < 0.05 versus the NC mimic-OE-NC group; #*p* < 0.05 versus the NC mimic-OE-Itpkb group. The measurement data were summarized as mean ± standard deviation. One-way ANOVA was carried out for comparisons among multiple groups followed by Tukey’s *post hoc* test. Cellular experiment was repeated three times independently.

To investigate whether miR-711 modulated the expression of inflammation-related factors and M2 transformation of BV2 cells through Itpkb, we first transfected miR-711 mimic into BV2 cells and infected them with lentivirus harboring OE-Itpkb at 24 h later. RT-qPCR revealed that OE-Itpkb treatment alone increased Itpkb expression in BV2 cells, while co-treatment of miR-711 mimic and OE-Itpkb caused an increase in miR-711 expression and a decrease in Itpkb expression (*p* < 0.01) (Figure 4A). Western blot assay showed that OE-Itpkb treatment alone increased Itpkb expression, while co-treatment of miR-711 mimic and OE-Itpkb resulted in decreased Itpkb expression (*p* < 0.01) (Figure 4B). ELISA confirmed that OE-Itpkb treatment alone caused increased expression of TNF-α and decreased expression of IL-10, while co-treatment of miR-711 mimic and OE-Itpkb resulted in decreased expression of TNF-α and increased expression of IL-10 (*p* < 0.01) (Figure 4C). Flow cytometry revealed that OE-Itpkb treatment alone decreased the ratio of M2/M1 in microglia while co-treatment of miR-711 mimic and OE-Itpkb increased the ratio of M2/M1 in microglia (*p* < 0.01) (Figure 4D). The above results suggest that miR-711 mediates the anti-inflammatory pathway and M2-like phenotype of microglia by targeting Itpkb.

### miR-711 Mediates Tau Protein Hyperphosphorylation via Itpkb Pathway

**FIGURE 5:**
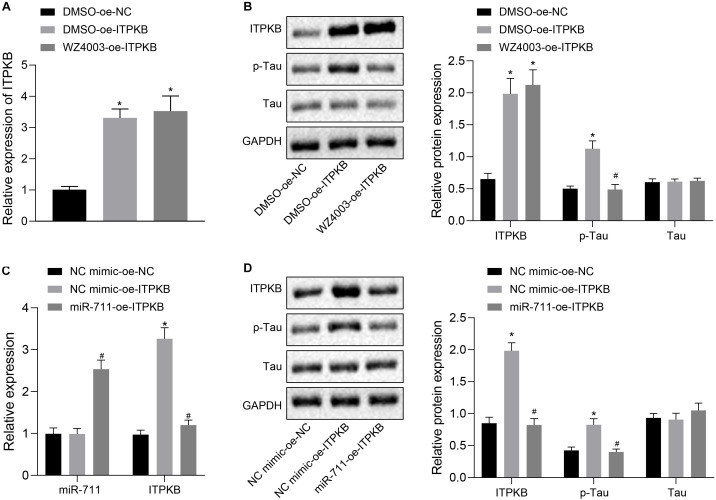
miR-711 mediates Tau protein hyperphosphorylation via Itpkb pathway. **(A)** RT-qPCR detection of Itpkb expression in each group of BV2 cells. **(B)** Western blot detection of Itpkb, p-Tau and Tau expression normalized to GAPDH in each group of BV2 cells. **(C)** RT-qPCR detection of miR-711 and Itpkb expression in each group of BV2 cells. **(D)** Western blot detection of the expression of Itpkb, p-Tau and Tau normalized to GAPDH in BV2 cells of each group. *p < 0.05 versus the NC mimic-OE-NC group or DMSO-OE-NC group; #*p* < 0.05 versus the DMSO-OE-Itpkb group or NC mimic-OE-Itpkb group. The measurement data were summarized as mean ± standard deviation. One-way ANOVA was carried out for comparisons among multiple groups followed by Tukey’s *post hoc* test. Cellular experiment was repeated three times independently.

To investigate whether miR-711 targeted Itpkb and modulated Tau hyperphosphorylation, we employed treatment with WZ4003, a Tau phosphorylation inhibitor. RT-qPCR detection showed that treatment with DMSO-OE-Itpkb promoted Iptkb expression in BV2 cells (*p* < 0.01), while Itpkb expression did not significantly differ between DMSO-OE-Itpkb and WZ4003-OE-Itpkb treatments (Figure 5A). Western blot analysis revealed that in BV2 cells, DMSO-OE-Itpkb increased Iptkb expression and Tau phosphorylation level, but did not affect the total Tau protein level. Relative to the DMSO-OE-Itpkb treatment, WZ4003-OE-Itpkb did not change the total Tau protein level, but decreased its phosphorylation level (*p* < 0.01) (Figure 5B). After verifying the inhibitory effect of WZ4003 on Tau phosphorylation, we transfected miR-711 mimic into BV2 cells and infected with OE-Itpkb lentivirus 24 h later. RT-qPCR detection revealed that in BV2 cells, OE-Itpkb alone caused an increase in Iptkb expression, while co-treatment of miR-711 mimic and OE-Itpkb caused an increase in miR-711 expression, and a reduction in Itpkb expression (*p* < 0.01) (Figure 5C). Western blot analysis revealed that in BV2 cells, treatment with OE-Itpkb alone stimulated an increase in Iptkb expression and raised the Tau phosphorylation level, but did not change the total Tau protein level. The co-treatment with miR-711 mimic and OE-Itpkb led to decreased Tau phosphorylation level (*p* < 0.01), but did not change the total Tau protein level (Figure 5D). The above results suggest that miR-711 mediates Tau protein hyperphosphorylation through the Itpkb pathway.

### miR-711-Loaded Microglia-Derived EVs Alleviate Inflammation, Neurodegeneration, and Cognitive Dysfunction in Mice by Targeting Itpkb

**FIGURE 6:**
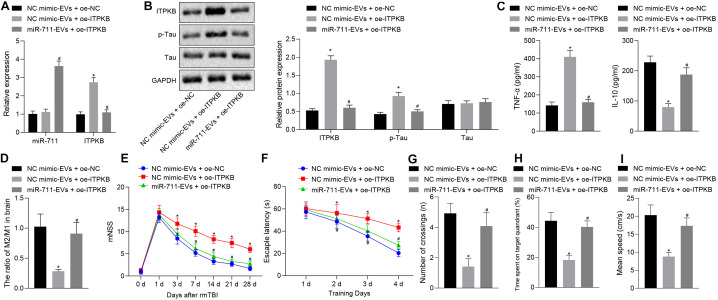
miR-711-loaded microglia-derived EVs alleviate inflammation, neurodegenerative changes and cognitive dysfunction in mice by targeting Itpkb. **(A)** RT-qPCR to detect the expression of miR-711 and Itpkb in the brain tissues of mice in each group. **(B)** Western blot assay to detect the expression of p-Tau and Itpkb protein in mouse brain tissues, normalized to GAPDH. **(C)** ELISA to detect the expression of inflammatory factor TNF-α and anti-inflammatory factor IL-10 in mouse serum. **(D**) Flow cytometry to detect the content of M2 phenotype and M1 phenotype microglia in mouse brain tissue, with the ratio calculated. **(E)** After the model was successfully constructed, the mice were scored for neurological deficits on days 1, 3, 7, 14, 21, and 28. **(F)** Quantitative analysis of the Morris water maze escape latency of the mice in each group during 4 days of training. **(G)** Quantitative analysis of the number of times that the mice of each group crossed the target platform. **(H)** Quantitative analysis of the percentage of time spent by the mice in the target quadrant. **(I)** Quantitative analysis of the average swimming speed of mice in each group. *p < 0.05 versus the NC mimic-EVs + OE-NC group; #*p* < 0.05 versus the NC mimic-EVs + OE-Itpkb group. The measurement data were summarized as mean ± standard deviation. One-way ANOVA was carried out for comparisons among multiple groups followed by Tukey’s *post hoc* test. Repeated measures ANOVA with Bonferroni *post hoc* test was employed to compare data at different time points. *n* = 12.

Following model development, rmTBI mice were injected with 40 μg BV2 cell-secreted EVs or lentivirus harboring overexpressed Itpkb via a tail vein. Then, 14 days later, the blood and brain tissues were collected. Analysis of brain tissues of rmTBI mice showed increased Itpkb expression in response to NC mimic-EVs + OE-Itpkb, while increased miR-711 and decreased Itpkb expression were observed in response to miR-711-EVs + OE-Itpkb (*p* < 0.01) (Figure 6A). Western blot assay revealed that Itpkb expression and p-Tau level increased in response to NC mimic-EVs + OE-Itpkb, but decreased in response to miR-711-EVs + OE-Itpkb treatment (*p* < 0.01) (Figure 6B). ELISA analysis of serum samples showed that TNF-α expression increased and IL-10 expression decreased in response to NC mimic-EVs + OE-Itpkb, but increased in response to miR-711-EVs + OE-Itpkb (*p* < 0.01) (Figure 6C). Flow cytometric analyses of single cell suspensions from mouse brain tissues showed that the ratio of microglia M2/M1 decreased in response to NC mimic-EVs + OE-Itpkb treatment, but decreased in response to miR-711-EVs + OE-Itpkb (*p* < 0.01) (Figure 6D). In addition, evaluation of neurological score changes in mice revealed that rmTBI mice had increased mNSS scores in the presence of NC mimic-EVs + OE-Itpkb, while miR-711-EVs + OE-Itpkb injection caused a decrease in mNSS score (*p* < 0.05) (Figure 6E). From the 28th day after rmTBI, the spatial navigation test and spatial probe test were employed to evaluate the spatial learning and memory abilities of mice for four consecutive days. There was a reduction in escape latency of all experimental groups with increasing number of swimming training days. The injection of NC mimic-EVs + OE-Itpkb prolonged the escape latency in rmTBI mice, while that of miR-711-EVs + OE-Itpkb shortened the escape latency (*p* < 0.05) (Figure 6F). During the spatial probe test, the number of times that the rmTBI mice traversed the target platform, the percentage of time spent in the target quadrant and the average swimming speed, were all reduced in the mice treated with NC mimic-EVs + OE-Itpkb. Conversely, treatment with miR-711-EVs + OE-Itpkb injection could increase the number of times crossing the target platform, the percentage of time spent in the target quadrant, and the average swimming speed (*p* < 0.01) (Figures 6G–I). The above results imply that microglia-derived EVs alleviate neurodegenerative changes and cognitive dysfunction in mice through miR-711-mediated Itpkb.

## Discussion

Microglia are the resident macrophages in brain, which participate in the neuroinflammatory pathways contributing to neurodegenerative alterations and cognitive dysfunction in AD (Crehan et al., 2012). Furthermore, EVs released by microglia can mediate the intercellular delivery of neurodegeneration-related Tau and β-amyloid in AD (Ge et al., 2020). Furthermore, miRNAs play a critical role in modulating microglial behavior and phenotype, and may prove useful as therapeutic biomarkers for AD and other neurodegenerative disorders (Freilich et al., 2013). In this study, we clarified that treatment with microglia-derived EVs loaded with miR-711, which mediated hyperphosphorylation of Tau protein in the Itpkb pathway, effectively alleviated neurodegenerative changes and cognitive dysfunction in AD.

The present findings indicated that miR-711 mediated the anti-inflammatory pathway and M2-like phenotype activation of microglia. A previous study has illuminated that miR-711 is highly expressed in M2 microglia, thus suggesting a role in anti-inflammatory pathways mediated by the M2-like phenotype (Freilich et al., 2013). However, the biological effects of miR-711 on microglia were not fully elaborated. The study of Redell et al. (2009) indicated the importance of fully characterizing the function and mechanism action of miR-711 deregulation, given its ability to modulate mRNA targets and biological processes involved in TBI pathophysiology. Emerging evidence has unmasked the potential of miRNAs in neuroinflammatory pathways and microglia activation; for example, ectoptic miR-155 expression results in neuroinflammation by enhancing generation of pro-inflammatory cytokines (Guedes et al., 2014). In the current study, miR-711 overexpression was observed to repress the production of pro-inflammatory TNF-α and augment anti-inflammatory IL-10, thus relieving neuroinflammation. Meanwhile, the elevated microglial M2/M1 ratio in response to miR-711 upregulation led to M2-like activation phenotype.

We further proceeded to illustrate the mechanism underlying the therapeutic role of miR-711 in rmTBI mice. Microglia-derived EVs relieved inflammation, neurodegeneration and cognitive dysfunction in the injured mice by delivering a cargo of miR-711. Being nano-sized vesicles, EVs have the ability to convey proteins and miRNAs from a source cell to target cells. The EVs shed by neurons are active factors in the interplay between glia cells and neurons, neurogenerative process, and neuroprotective molecule transportation (Hill, 2019; Upadhya et al., 2020). It is interesting to note that delivery of EVs to neurons has been suggested to be orchestrated by microglia, where specific miRNAs carried by microglial EVs can play a significant role in synaptic plasticity, learning and memory, and neuroinflammation (Brites and Fernandes, 2015). Since aforementioned results of our study confirmed the function of ectopic miR-711 in neuroinflammation and activation of the M2 like phenotype, it is reasonable to suppose that miR-711-containing EVs from microglia attenuated neurodegeneration and cognitive decline in the present model of AD.

Moreover, our mechanistic exploration identified a binding affinity between miR-711 and Itpkb. Specifically, miR-711 targeted and negatively regulated Itpkb. A prior study by Saetre et al. (2011) has documented the implication of Itpkb in neuronal intracellular calcium signaling pathway, which is a biological signaling associated with AD. Upregulation of Itpkb has been highlighted in cerebral cortex from patients affected by AD, where it evidently contributes to neuronal apoptosis and exacerbation of AD pathology (Stygelbout et al., 2014). Furthermore, the promoting effect of Itpkb in AD model has been revealed to correlate with the regulation of miR-132 expression, which also targeted and inversely modulated Itpkb (Salta et al., 2016). Corroborating findings in our study demonstrated that miR-711 mediated an anti-inflammatory pathway of microglia of the M2-like phenotype by targeting Itpkb. Furthermore, we documented in our study the involvement of Tau phosphorylation. Notably, miR-711 mediated an increasing in Tau protein hyperphosphorylation via the Itpkb pathway. Abnormal hyperphosphorylation of Tau protein has already been manifested in AD associated with neurological deficits (Xia et al., 2017; Franzmeier et al., 2020). Meanwhile, research shows that EVs derived from AD brain samples can shed Tau protein (Muraoka et al., 2020). The aberrant Tau hyperphosphorylation in AD might be governed by treatment with EVs, thus impeding the progression of AD (Perrotte et al., 2020). Indeed, the present experiments *in vivo* substantiated that miR-711-loaded microglia-derived EVs alleviated inflammation, neurodegeneration, and cognitive dysfunction in the model mice by targeting Itpkb.

Taken together, microglia-derived EVs transfer miR-711 into neurons, inhibiting the expression of Itpkb and thereby inhibiting the Itpkb-induced hyperphosphorylation of Tau, a mechanism of which relieves inflammation, neurodegeneration and cognitive dysfunction in the present mouse repetitive head injury model AD. Based on the present findings, we suggest that therapeutic strategies for AD should be directed toward the artificial overexpression of miR-711, which may potentially present a clinically viable target for attenuating neuroinflammation and cognitive decline.

## Data Availability Statement

The original contributions presented in the study are included in the article/supplementary material, further inquiries can be directed to the corresponding author.

## Ethics Statement

The animal study was reviewed and approved by Animal Ethics Committee of Jilin University.

## Author Contributions

SN, YZ, and CX wrote the main manuscript text. YN collected the data. SN prepared all figures. All authors reviewed the manuscript.

## Conflict of Interest

The authors declare that the research was conducted in the absence of any commercial or financial relationships that could be construed as a potential conflict of interest.

## Footnotes

1 http://www.targetscan.org/vert_71/

2 http://www.bioconductor.org/packages/release/bioc/html/limma.html

3 https://www.ncbi.nlm.nih.gov/gds

4 https://string-db.org/

5 https://cytoscape.org/
